# An Engineered Cardiac Reporter Cell Line Identifies Human Embryonic Stem Cell-Derived Myocardial Precursors

**DOI:** 10.1371/journal.pone.0016004

**Published:** 2011-01-04

**Authors:** Carissa Ritner, Sharon S. Y. Wong, Frank W. King, Shirley S. Mihardja, Walter Liszewski, David J. Erle, Randall J. Lee, Harold S. Bernstein

**Affiliations:** 1 Cardiovascular Research Institute, University of California San Francisco, San Francisco, California, United States of America; 2 Department of Medicine, University of California San Francisco, San Francisco, California, United States of America; 3 Eli and Edythe Broad Center of Regeneration Medicine and Stem Cell Research, University of California San Francisco, San Francisco, California, United States of America; 4 Department of Pediatrics, University of California San Francisco, San Francisco, California, United States of America; Brigham and Women's Hospital, United States of America

## Abstract

Unlike some organs, the heart is unable to repair itself after injury. Human embryonic stem cells (hESCs) grow and divide indefinitely while maintaining the potential to develop into many tissues of the body. As such, they provide an unprecedented opportunity to treat human diseases characterized by tissue loss. We have identified early myocardial precursors derived from hESCs (hMPs) using an α-myosin heavy chain (αMHC)-GFP reporter line. We have demonstrated by immunocytochemistry and quantitative real-time PCR (qPCR) that reporter activation is restricted to hESC-derived cardiomyocytes (CMs) differentiated *in vitro*, and that hMPs give rise exclusively to muscle in an *in vivo* teratoma formation assay. We also demonstrate that the reporter does not interfere with hESC genomic stability. Importantly, we show that hMPs give rise to atrial, ventricular and specialized conduction CM subtypes by qPCR and microelectrode array analysis. Expression profiling of hMPs over the course of differentiation implicate Wnt and transforming growth factor-β signaling pathways in CM development. The identification of hMPs using this αMHC-GFP reporter line will provide important insight into the pathways regulating human myocardial development, and may provide a novel therapeutic reagent for the treatment of cardiac disease.

## Introduction

Over five million people in the United States alone suffer with heart failure [1] because unlike some organs, the heart is unable to repair itself after injury [2]. Human embryonic stem cells (hESCs) grow and divide indefinitely while maintaining the potential to develop into many tissues of the body. As such, they provide an unprecedented opportunity to treat a variety of human diseases characterized by tissue loss or insufficiency. Animal studies have shown that pluripotent hESCs have a high risk of tumor formation [3], while fully differentiated hESC-derived cardiomyocytes (CMs) confer only modest functional benefit [4]. This suggests that from a developmental standpoint, mature CMs may be beyond the ability to fully incorporate into existing muscle. Therefore, the identification of hESC-derived myocardial precursors that are committed to the cardiac lineage, but retain the plasticity to facilitate complete engraftment has been an important goal [5].

Work over the past decade has shown that hESCs differentiate into a heterogeneous population of CMs in culture, with gene expression patterns and electrophysiological properties reminiscent of embryonic atrium, ventricle and specialized conduction tissue [6], [7], [8], [9], [10]. The mechanisms that drive CM subtype specification, however, are not well understood.

To approach both the need for a model system with which to elucidate the process of human CM subtype specification, and provide a source of human myocardial precursors for cell therapy studies, we engineered a hESC line that identifies multipotent myocardial precursor (hMP) cells. These cells give rise to multiple CM subtypes, and is therefore uniquely suited to address both of these needs.

## Results

### Construction of an α-myosin heavy chain human embryonic stem cell reporter line

**Figure 1:**
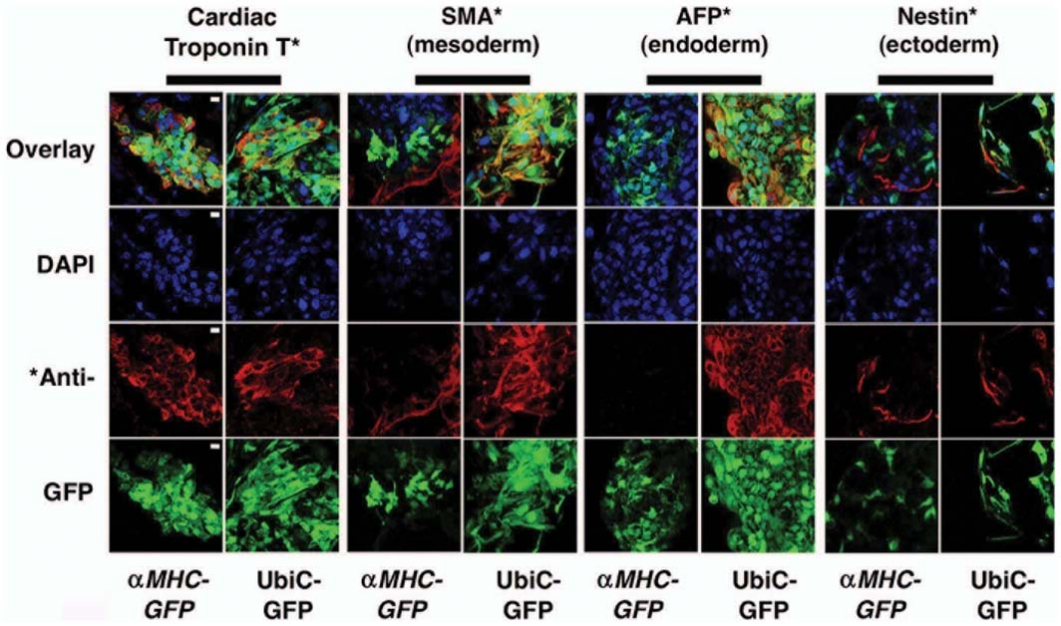
The αMHC-GFP reporter line is cardiac-specific. hESCs from either the αMHC-GFP or UbiC-GFP reporter lines were differentiated for 14 days by hEB formation, and analyzed by direct and indirect immunofluorescence for expression of GFP and cTnT, SMA, AFP or nestin, respectively. Cells were counterstained with DAPI to identify nuclei. Typical micrographs are shown. Co-localization of GFP solely with cardiac troponin T, but not SMA, AFP or nestin, was seen in differentiated αMHC-GFP hESCs. In contrast, co-localization of GFP with all four markers was seen in differentiated UbiC-GFP hESCs. Bar, 100 µm.

The α-myosin heavy chain (αMHC) gene has been shown to be expressed both early and late during murine cardiac development [11], [12]. Taking advantage of this developmentally broad expression pattern, we used a subsequence of the murine αMHC promoter consisting of nucleotides −1679 through +1 relative to the translation start site of the αMHC mRNA to construct a myocardial-specific enhanced GFP reporter in the H9 hESC line. This contains MEF1 and MEF2 binding sites, two thyroid hormone response elements, the cardiac troponin T-responsive MCAT element, and SRF binding motif [13]. We used the HIV-derived, self-inactivating lentiviral vector, 2K7_bsd_ [14], in which the HIV *gag*, *pol*, and *env* genes are deleted and the HIV-1 flap sequence and woodchuck hepatitis virus post-transcriptional regulatory element are included to improve infectious titer and gene expression. To mitigate against the effects of clonal variation, we isolated stable transfectants by population selection. Presumably, cells in which viral integration disrupted essential genes did not survive selection. Upon differentiation of the resulting αMHC-GFP hESC line by human embryoid body (hEB) formation, GFP expression was detected solely in 14 day hEBs co-expressing cardiac troponin T (cTnT) (**Fig. 1**).

### The αMHC-GFP reporter is activated solely in hESC-derived cardiomyocytes

To specifically localize cardiac versus non-cardiac proteins within differentiating αMHC-GFP^+^ hEBs, and confirm that GFP did not inhibit the expression of non-cardiac markers, we determined co-localization of alpha-fetoprotein, a marker of primitive endoderm, nestin, a marker of neuroectoderm, and smooth muscle actin, a marker of non-cardiac mesoderm, with GFP in the reporter line, compared to the co-localization of these proteins with ubiquitin C-driven, constitutive expression of GFP in a ubiC-GFP hESC line at day 14 of hEB differerentiation. This demonstrated that while CM-specific cTnT, but not smooth muscle actin, alpha-fetoprotein or nestin, co-localized with αMHC-driven GFP expression in the αMHC-GFP reporter line, all of these markers co-localized with ubiquitin C-driven GFP expression in the ubiC-GFP line (**Fig. 1**). This also confirmed that the absence of smooth muscle actin, alpha-fetoprotein or nestin co-expression with αMHC-GFP was not due to interference by GFP expression.

**Figure 2:**
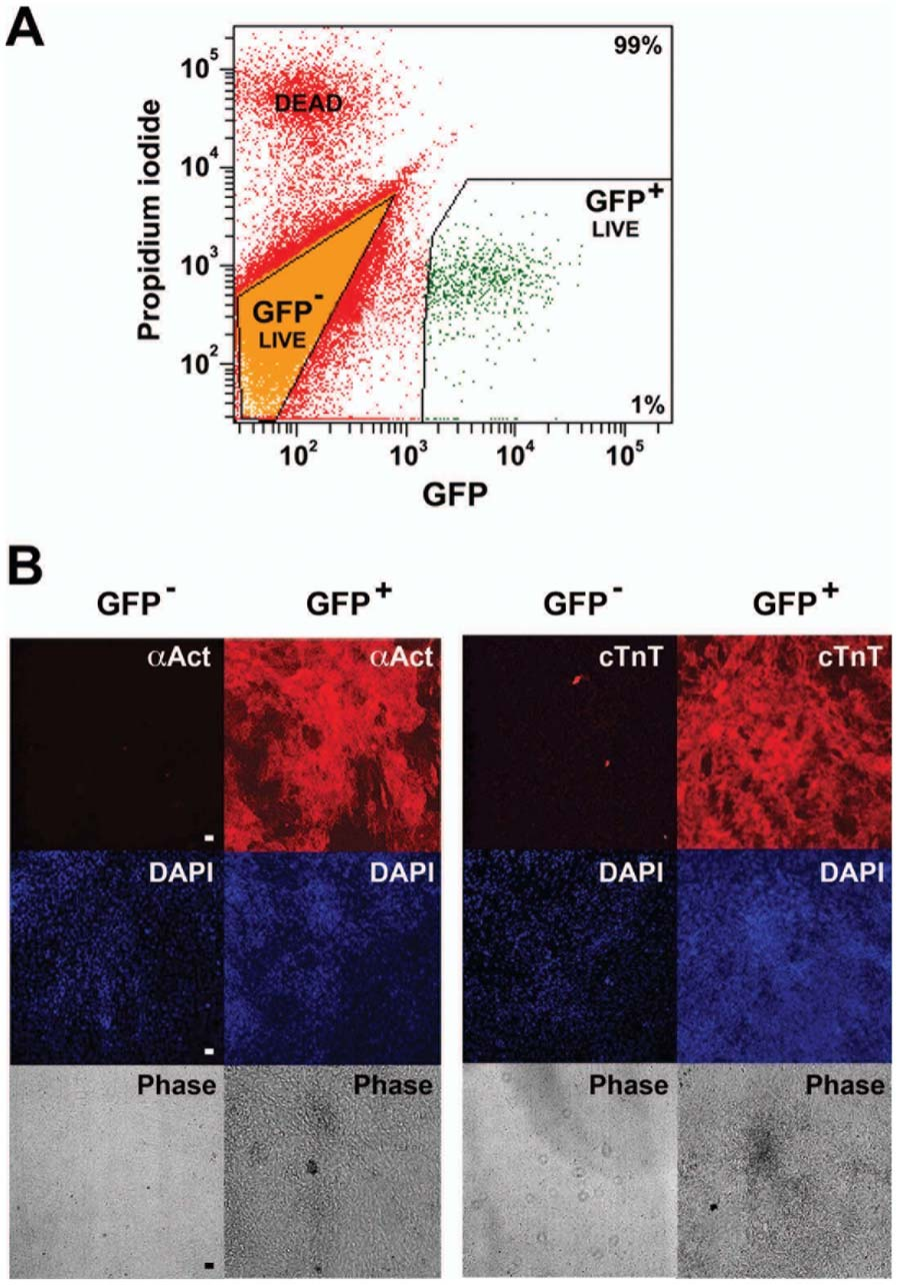
Sorted αMHC-GFP^+^ hESCs form CMs *in vitro*. αMHC-GFP hESCs were cultured under differentiation conditions for 8 days. (A) Day 8 hEBs were suspended as single cells and sorted for GFP expression. Propidium iodide was used to discriminate live versus dead cells. A typical sort is shown. (B) GFP^+^ cells were re-cultured under differentiation conditions for an additional 6 days, then analyzed *in situ* by indirect immunofluorescence for expression of cTnT and cardiac α-actinin. Cells were counterstained with DAPI to identify nuclei. Typical micrographs are shown. Expression of cTnT and cardiac α-actinin was seen throughout the culture, which demonstrated multiple foci of spontaneous contractile activity (**Supplementary Videos 1 and 2**). Bar, 400 µm.

To determine the fate of selected αMHC-GFP^+^ hEBs, we sorted hEBs for GFP expression at day 8 of differentiation, then re-cultured αMHC-GFP^+^ cells under differentiation conditions for an additional 6 days (day 14 of hEB differentiation) and assessed CM-specific cTnT and cardiac α-actinin expression by *in situ* immunocytochemistry. This showed that cells activating the reporter on day 8 generated a lawn of cTnT^+^ cardiac α-actinin^+^ CMs in culture (**Fig. 2**), and that these cells demonstrate spontaneous contractile activity (**Movie S1 and S2**).

### αMHC-GFP-derived myocardial precursors give rise to muscle *in vivo*

**Figure 3:**
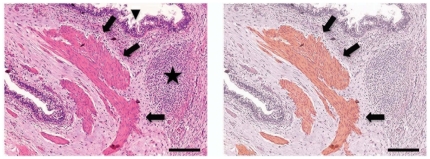
hMPs form muscle *in vivo*. Teratomas formed from αMHC-GFP hESCs by renal capsule grafting in SCID mice were analyzed by hematoxylin and eosin staining to identify tissue structures (*left*), and immunohistochemistry with anti-GFP antibody (*right*) to identify tissues arising from hMPs. Section from typical teratoma shown (n = 7). Primitive neural epithelium (star) and glandular intestinal structures (arrowheads) can be seen surrounding striated muscle (arrows) (*left*), which stains positive for GFP expression (brown; *right*). GFP was expressed exclusively in mononucleated myofibers within the teratomas, and was not found in any other tissues (not shown). Bar, 100 µm.

To establish that hESCs expressing the αMHC-GFP reporter also give rise to muscle *in vivo*, we evaluated undifferentiated αMHC-GFP hESCs by teratoma assay. We grafted 10^6^ hESCs beneath the kidney capsule of 8-week-old SCID mice, and analyzed for teratoma formation after 10 weeks. The αMHC-GFP hESCs consisted of tissues arising from all three embryonic germ layers, however, indirect immunohistochemical analysis showed that GFP expression was restricted to striated muscle, specifically mononucleated myofibers characteristic of cardiac muscle (**Fig. 3**). Restricted GFP localization to cardiac muscle in all teratomas analyzed also suggested that hESCs expressing the αMHC-GFP reporter do not form tumors comprised of other tissues.

### αMHC-GFP hESCs remain genetically stable

**Figure 4:**
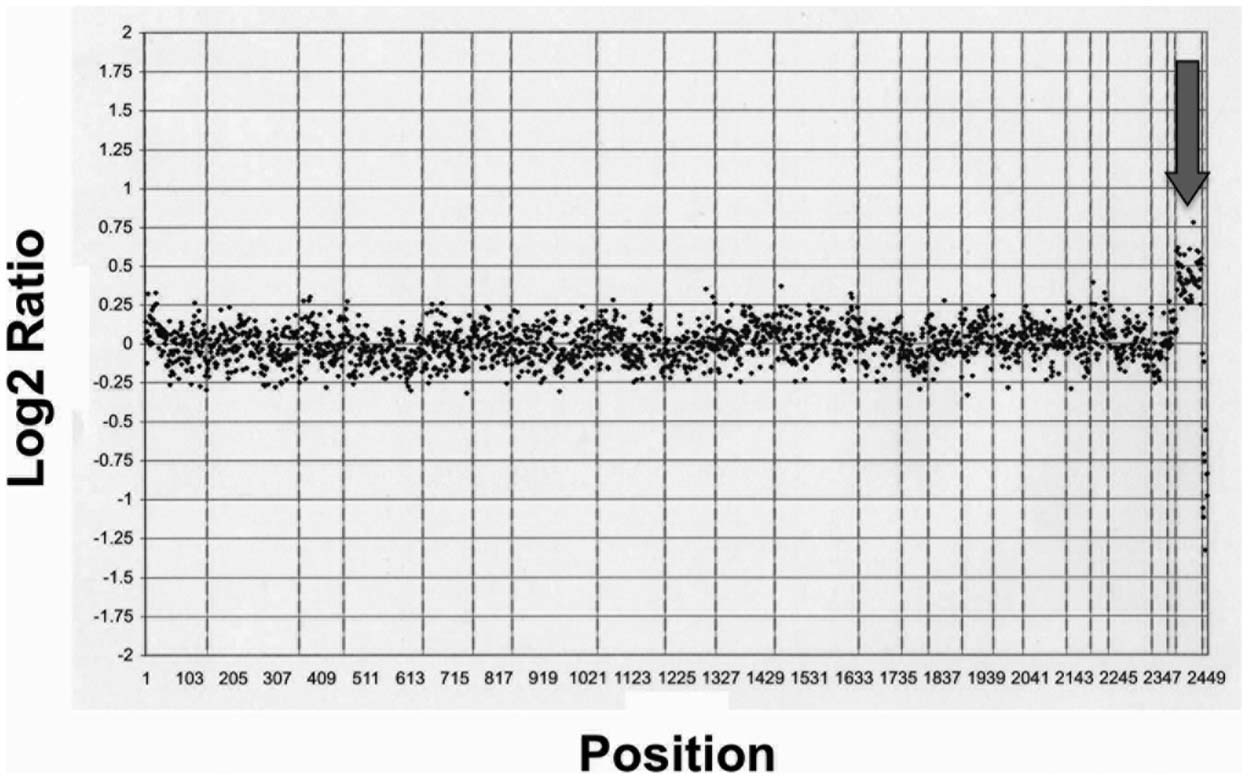
The αMHC-GFP reporter line maintains genomic integrity. αMHC-GFP hESCs (H9; 46,XX) passaged ten times after stable selection were analyzed by array comparative genomic hybridization. The log_2_ ratios for all loci on the array were plotted in genome order from chromosome 1pter to Xqter. The data were normalized so that the log_2_ ratio = 0 for genomic regions that are present in diploid copy number. Male reference DNA was used in the hybridization as indicated by the sex mismatch for chromosome X (arrow). There was no evidence of copy loss or gain, consistent with maintenance of euploidy.

It is well-established that hESCs maintained in culture are at risk for developing aneuploidy [15], [16]. Therefore, it becomes especially important to confirm genomic stability in any hESC-derived line to be used for either developmental investigation or therapy. To assess the genomic integrity of the αMHC-GFP hESC line, we analyzed cells for single chromosomal region gains and losses by array comparative genomic hybridization [17]. This showed that αMHC-GFP hESCs maintain a normal karyotype at the level of individual gene regions (**Fig. 4**).

### The αMHC-GFP reporter identifies an early myocardial precursor that gives rise to multiple CM subtypes

**Figure 5:**
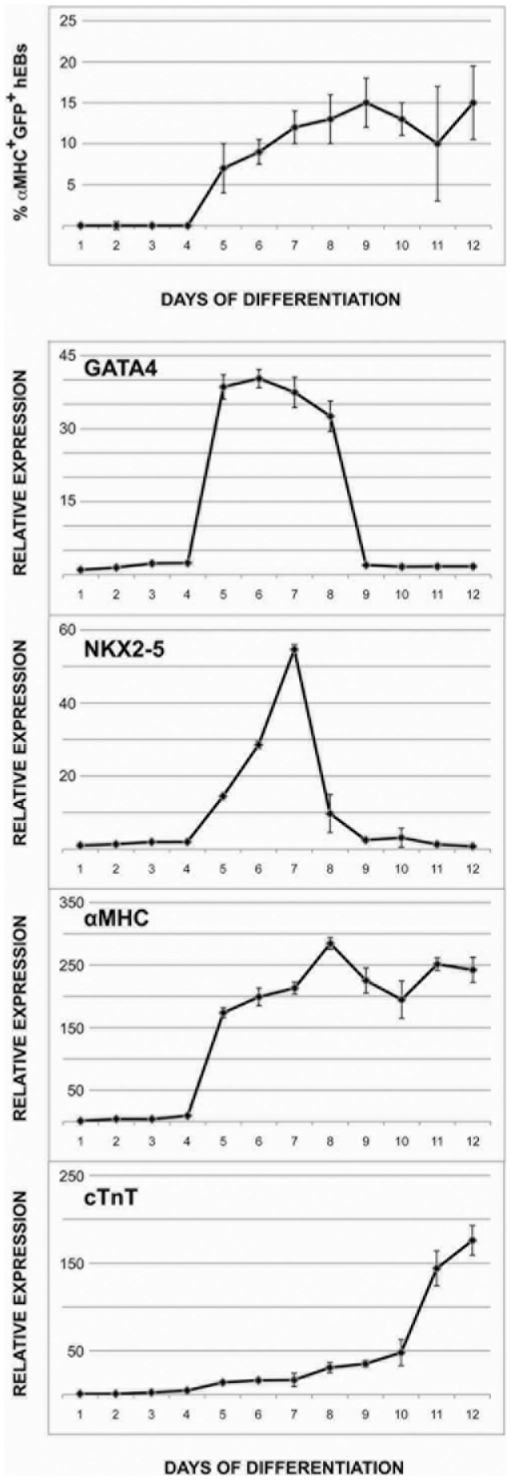
Reporter activation coincides with early cardiogenesis. αMHC-GFP hESCs were differentiated for 12 days and the number of hMPs (GFP^+^αMHC^+^) cells were scored as a percentage of total hESC number on each day. On days 1–4, before the onset of GFP expression, unsorted differentiating hESCs were analyzed by qPCR. On days 5–12, sorted GFP^+^ hMPs were analyzed by qPCR for expression of cTnT, NKX2-5, GATA4 and αMHC, relative to undifferentiated hESCs at day 1. Data shown represent mean±s.e.m. (N = 3). Reporter activation was first observed on day 5, with 15% GFP^+^ (αMHC^+^) hESCs seen by day 9 (*top*). Endogenous αMHC, GATA4 and NKX2-5 expression was observed with activation of the reporter on day 5, while strong expression of cTnT did not appear until day 11 (*bottom*).

Others have shown that CMs derived from hESCs in culture can display the molecular and electrical properties of embryonic ventricular, atrial and nodal tissue [6]. This heterogeneity occurs with differentiation in culture using a variety of conditions [7], [8], [9], [10], and suggests that hESCs are capable of giving rise to myocardial precusors that precede the developmental branchpoint between first and second heart field specification. We counted the number of GFP^+^ (αMHC^+^) hESCs over the course of hEB differentiation, and found that these cells started to appear in culture at day 5, commensurate with the onset of GATA4 and NKX2-5 expression, and several days before the expression of cTnT, which is associated with spontaneous contractile activity [18] (**Fig. 5**).

**Figure 6:**
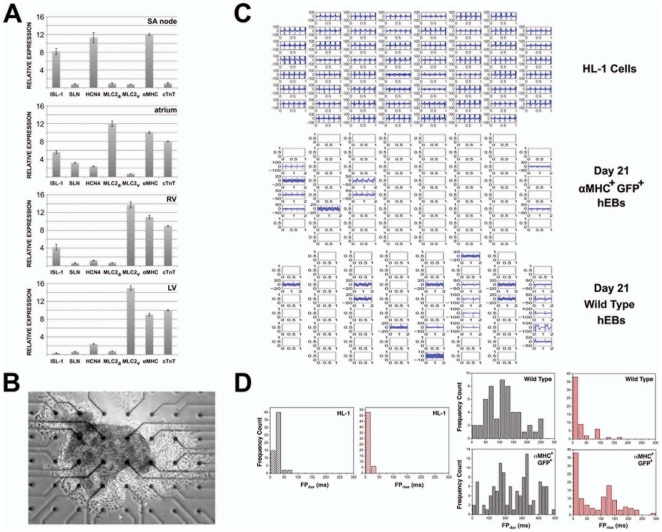
hMPs give rise to multiple CM subtypes. αMHC-GFP or wild type hESCs were differentiated for 21 days, and beating GFP^+^ or wild type hEBs were manually dissected for further analysis. (A) GFP^+^ hEBs were analyzed for expression of Islet-1 (ISL-1), sarcolipin (SLN), Na^+^/K^+^ hyperpolarization-activated cyclic nucleotide-gated channel 4 (HCN4), myosin light chain-2 atrial (MLC2a), MLC2v, αMHC and cTnT, relative to undifferentiated hESCs. Gene expression patterns consistent with embryonic atrium, sinoatrial (SA) node, left (LV) and right (RV) ventricle were seen. Data shown represent mean±s.e.m. for at least three hEBs with similar expression profiles for these seven genes. (B) A typical GFP^+^ hEB cultured on a gelatin-coated microelectrode array for 2 days is shown. (C) Sample field potential (FP) tracings recorded at arrayed microelectrodes are shown for HL-1 cells and typical αMHC-GFP^+^ or wild type hEBs. (D) Field potentials of averaged HL-1 cultures or hEBs were analyzed, and the number of tracings with a given FP duration (FP_dur_) or decay of extracellular potential (FP_rise_) were plotted as a function FP_dur_ (*left*) or FP_rise_ (*right*). Compared to the homogeneous FP recordings of HL-1 cultures, both αMHC-GFP^+^ (n = 7) and wild type (n = 5) hEBs showed similarly heterogeneous, averaged FP recordings, consistent with a heterogeneous distribution of action potential durations extracellular potential decay.

Since the reporter appeared to identify early human myocardial precursors (hMPs), we wanted to determine whether these hMPs were capable of generating a heterogeneous population of human CMs based on molecular and electrical characteristics. We differentiated αMHC-GFP hESCs for 21 days, and manually dissected GFP^+^ hEBs for analysis by qPCR and microelectrode array. Evaluation of individual GFP^+^ hEBs by qPCR demonstrated that molecular signatures characteristic of embryonic atrial (Islet-1 (ISL-1)^+^ sarcolipin (SLN)^+^ Na^+^/K^+^ hyperpolarization-activated cyclic nucleotide-gated channel 4 (HCN4)^−^ myosin light chain-2 atrial (MLC2a)^+^ MLC2v^−^ αMHC^+^ cTnT^+^), left (ISL-1^−^ SLN^−^ HCN4^−^ MLC2a^−^ MLC2v^+^ αMHC^+^ cTnT^+^) and right (ISL-1^+^ SLN^−^ HCN4^−^ MLC2a^−^ MLC2v^+^ αMHC^+^ cTnT^+^) ventricular, and nodal (ISL-1^+^ SLN^−^ HCN4^+^ MLC2a^−^ MLC2v^−^ αMHC^+^ cTnT^−^) tissue were present in distinct hEBs (**Fig. 6A**) at an approximate ratio of 40% ISL-1^+^ SLN^+^ HCN4^−^ MLC2a^+^ MLC2v^−^ αMHC^+^ cTnT^+^, 10% ISL-1^+^ SLN^−^ HCN4^+^ MLC2a^−^ MLC2v^−^ αMHC^+^ cTnT^−^, 30% ISL-1^+^ SLN^−^ HCN4^−^ MLC2a^−^ MLC2v^+^ αMHC^+^ cTnT^+^ and 20% ISL-1^−^ SLN^−^ HCN4^−^ MLC2a^−^ MLC2v^+^ αMHC^+^ cTnT^+^ among >30 hEBs analyzed.

To determine whether the electrical properties of GFP^+^ hEBs were similarly heterogeneous in nature, we allowed dissected hEBs to adhere to microelectrode arrays (MEAs) (**Fig. 6B**) and performed simultaneous recording of extracellular field potentials (FP) over time (**Fig. 6C**). This allowed us to measure the size of the largest negative peak (FP_min_), the last postive peak of the cycle (FP_max_), the time interval between FP_min_ and FP_max_ (FP_dur_), and decay of the extracellular potential (time from the onset of the FP to FP_min_ = FP_rise_). Others have shown that FP_rise_ directly correlates to action potential rise time, and FP_dur_ directly correlates to action potential duration [19], [20]. Compared to HL-1 cells, which represent a homogenous culture of mouse atrial CMs [21] with FP_dur_≤75 ms and FP_rise_≤50 ms, both αMHC-GFP^+^ and wild type, beating hEBs demonstrated similarly heterogeneous combinations of FP_dur_ (25–250 ms versus 10–475 ms; *p* = 0.86) and FP_rise_ (10–175 ms versus 10–275 ms; *p* = 0.83) (**Fig. 6D**).

### Expression profiling of human CM differentiation

**Figure 7:**
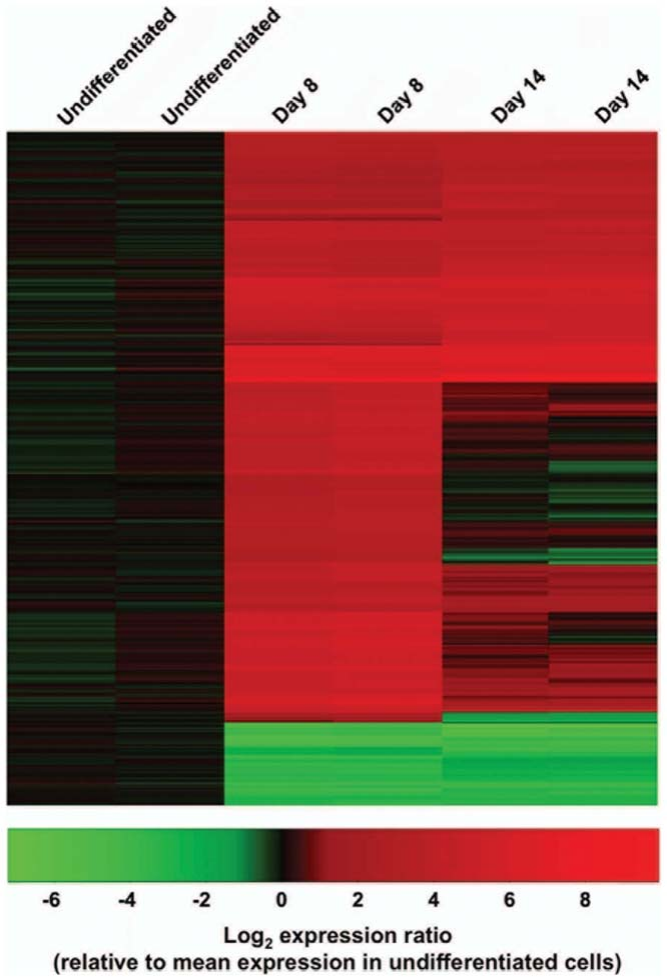
Expression profiling of differentiating hMPs. Relative gene expression in undifferentiated αMHC-GFP hESCs, hMPs sorted from day 8 αMHC-GFP hEBs, and hMPs sorted from day 14 αMHC-GFP hEBs (two samples each) was analyzed. Heat map shows log_2_-fold change (sample intensity/mean intensity for undifferentiated group) for the 830 genes that had statistically significant differences (false discovery rate (FDR) <0.05) of at least 2-fold in any pairwise comparison between the three groups.

Since we had established that this αMHC-GFP hESC line could identify myocardial precusors capable of giving rise to all embryonic CM subtypes, we used it to examine potential pathways involved in CM differentiation. We isolated hMPs at day 8 of differentiation, after the onset of GATA4, NKX2-5 and αMHC expression, but before cTnT expression or spontaneous contractions (**Fig. 5**), and at day 14 of differentiation, after the onset of cTnT expression and spontaneous beating (**Fig. 1**), and compared the gene expression profiles of these two differentiation time points to each other and to undifferentiated hESCs using an array of >41,000 unique sequences representing the whole human genome. Analysis of the 830 most highly differentially expressed genes from biological replicates of each time point resulted in the clustering shown in **Fig. 7**. This indicated that the reproducibility of hMP differentiation was reasonably robust. Overall, there were 3,279 probes targeting human genes that showed differential expression across the three time points with a false discovery rate <0.05. The complete data set has been deposited in Gene Expression Omnibus (http://www.ncbi.nlm.nih.gov/geo/; Accession Number GPL6480).

Analysis of cardiac-specific gene expression demonstrated that hMP differentiation proceeded in a manner commensurate with cardiac morphological development (**Table 1**). Differentiating hMPs expressed markers of cardiac tube formation (NKX2-5, GATA4) and looping (MEF2c, αMHC) by day 8, and markers of spontaneous contractile activity (cTnT, cTnI) by day 14. In addition, expression levels of atrial (ISL-1, ANF) and right ventricular (ISL-1, HAND2) genes were observed by day 8 and continued to rise through day 14, while expression of genes associated with development of the atrioventricular septum (TBX2) and left ventricle (HAND1, TBX5) were observed by day 14.

**Table 1:** Fold-changes in Cardiac-Specific Genes.

Cardiac Genes	Stage/Chamber*	Day 8 v. Day 0 *[FDR* ** *]*		Day 14 v. Day 0 *[FDR]*	
NKX2-5	*tube*	34	*[0.002]*	42	*[0.001]*
GATA4	*tube*	24	*[0]*	29	*[0]*
MEF2c	*loop*	28	*[0]*	26	*[0]*
αMHC	*loop*	841	*[0]*	886	*[0]*
cTnT	*contract*	-	-	122	*[0]*
cTnI	*contract*	-	-	32	*[0]*
ISL-1	*A/RV*	4	*[0.012]*	16	*[0.001]*
ANF	*A*	82	*[0]*	317	*[0]*
HAND2	*RV*	232	*[0]*	476	*[0]*
TBX2	*AV*	-	-	46	*[0]*
HAND1	*LV*	-	-	33	*[0]*
TBX5	*LV*	-	-	74	*[0]*

*A, atrium; RV, right ventricle; AV, atrioventricular septum; LV, left ventricle.

**FDR, false discovery rate.

The role of Wnt signaling during myocardial specification and early cardiac morphogenesis has been described [22]. Wnt ligands and Frizzled receptors specifically have been implicated during early heart development. This is supported by our findings that the expression of Wnt1, 2, 5B and 11, as well as Frizzled 4 and 5 was upregulated by day 8 of hMP differentiation (**Table 2**). In addition, secreted Frizzled-related proteins (SFRPs) that are known to inhibit Wnt signaling and regulate CM proliferation, were downregulated at day 8, but then upregulated in the case of SFRP3 at day 14 (**Table 2**). CM migration during heart development has been shown to rely on transforming growth factor β (TGFβ) signaling [23]. Consistent with its role in cardiogenesis, several effectors and inhibitors of TGFβ pathways were upregulated and downregulated, respectively, over the course of hMP differentiation (**Table 3**).

**Table 2:** Fold-changes in Wnt Pathway Genes.

*Wnt Genes*	Day 8 v. Day 0 *[FDR]* *		Day 14 v. Day 0 *[FDR]*	
*Effectors*				
Wnt1	17	*[0.001]*	32	*[0]*
Wnt2	47	*[0]*	26	*[0]*
Wnt2B	-	*-*	3	*[0.039]*
Wnt5A	-	*-*	5	*[0.009]*
Wnt5B	4	*[0.005]*	5	*[0.002]*
Wnt8B	-	*-*	4	*[0.041]*
Wnt11	8	*[0.007]*	10	*[0.005]*
Activin A receptor	4	*[0.011]*	4	*[0.007]*
N-cadherin 2	4	*[0.042]*	-	*-*
DKK2	-	*-*	5	*[0.022]*
DKK3	5	*[0.002]*	7	*[0.001]*
Frizzled 4	4	*[0.018]*	-	*-*
Frizzled 9	5	*[0.012]*	-	*-*
Frizzled 10	-	*-*	3	*[0.017]*
LEF1	15	*[0.001]*	20	*[0]*
NFATC2	4	*[0.004]*	5	*[0.002]*
NFATC4	-	*-*	3	*[0.011]*
RARβ	-	*-*	3	*[0.045]*
*Inhibitors*				
PPP2CA	−2	*[0.045]*	−6	*[0.046]*
PPP2R1B	−5	*[0.013]*	−4	*[0.027]*
PPP2R2B	−9	*[0.002]*	−6	*[0.003]*
SFRP1	−3	*[0.017]*	-	*-*
SFRP2	−19	*[0.006]*	−7	*[0.001]*
SFRP3	-	*-*	5	*[0.029]*
SOX2	−17	*[0.001]*	−9	*[0.002]*
SOX3	−12	*[0.003]*	−5	*[0.016]*
SOX11	−3	*[0.018]*	-	*-*

*FDR, false discovery rate.

**Table 3:** Fold-changes in TGFβ Pathway Genes.

*TGFβ Genes*	Day 8 v. Day 0 *[FDR* * *]*		Day 14 v. Day 0 *[FDR]*	
*Effectors*				
TGFβ2	31	*[0]*	51	*[0]*
TGFβ3	4	*[0.043]*	-	*-*
Activin/Inhibin A	3	*[0.013]*	-	*-*
Activin A receptor	4	*[0.011]*	4	*[0.007]*
BMP2	16	*[0]*	18	*[0]*
BMP4	6	*[0.013]*	25	*[0.001]*
BMP5	65	*[0]*	135	*[0]*
BMP7	8	*[0.003]*	10	*[0.002]*
BMP10	14	*[0.013]*	-	*-*
SMAD3	-	*-*	2	*[0.022]*
SMAD6	4	*[0.019]*	-	*-*
SMAD9	4	*[0.007]*	8	*[0.001]*
Id2	7	*[0.003]*	-	*-*
Id3	4	*[0.012]*	-	*-*
Id4	12	*[0.001]*	-	*-*
*Inhibitors*				
MAP3K7	−2	*[0.038]*	-	*-*
MAPK1	−3	*[0.038]*	-	*-*
MAPK8	−2	*[0.027]*	-	*-*
c-Myc	−13	*[0.001]*	−10	*[0.001]*
DP1	−2	*[0.050]*	−3	*[0.017]*
E2F4	−3	*[0.028]*	−3	*[0.020]*

*FDR, false discovery rate.

## Discussion

Although β-myosin heavy chain (βMHC) is the predominant isoform expressed during murine embryonic and fetal development, the transition from βMHC to αMHC begins as early as day 7.5 p.c. in mouse embryos with the appearance of somites and the onset of cardiac tube formation [11], [12]. Expression of both isoforms approaches equivalence as the cardiac tube begins to contract by day 9.5 p.c., and the β/α ratio begins to reverse with αMHC becoming the predominant isoform during postnatal life [11], [12]. These observations during mouse development *in vivo* parallel mouse ESC (mESC) differentiation *in vitro*. αMHC is expressed by day 8 of mESC differentiation and is expressed exclusively in beating mouse EBs [12]. While many studies have used the murine αMHC promoter to track mature CMs, our data suggest that because of its expression across early and mature stages of CM development even between species, the murine αMHC promoter can be used to track CM differentiation from a multipotent myocardial precursor in real time. This has afforded us an unprecedented opportunity to study human CM differentiation from undifferentiated ESCs through EB formation and ultimately embryonic CM subtypes.

Other laboratories have developed transgenic/reporter hESC lines to derive differentiated CMs. As distinct from the work described here, however, these lines have allowed either for the selection of mature CMs only, or of progenitors that give rise to non-muscle cardiac cells in addition to myocardial cells. Huber *et al.* used lentiviral vectors to produce stable hESC lines in which enhanced GFP was expressed under control of the MLC2v promoter [24]. While these lines were able to generate electrically active CMs of unspecified subtype, MLC2v is expressed later in CM differentiation than αMHC, and does not afford the same insight into early CM differentiation. Xu *et al.* generated stable hESC lines using a plasmid containing the αMHC promoter driving expression of the neomycin resistance gene [25]. While this afforded an effective strategy for enriching CMs for cell transplant experiments, it does not accommodate the study of developmental stages over time. Kita-Matsuo *et al.* recently reported the design of a set of lentiviral vectors to generate multiple stable hESC lines with eGFP and mCherry reporters or with puromycin resistance using the αMHC promoter [26]. The focus of these studies was to create tools to enhance CM production for large-scale clinical application. While these investigators demonstrated that hESC-derived CMs have gene expression profiles similar to those found in adult hearts and electrophysiological properties of embryonic CMs, analysis of CM differentiation using these lines was not reported. To track the fate of human Isl1^+^ cells and their progeny during hESC differentiation, Bu *et al.* used Isl1:*cre* hESCs transfected with a pCAG-flox-DsRed reporter plasmid to achieve irreversible DsRed expression in Isl1^+^ cells. In clonal assays of day 8 hEBs, about half of the DsRed^+^ clones expressed markers of the three major cardiac lineages, cTnT (CMs), PECAM1/CD31 (endothelial cells), and smooth muscle troponin (smooth muscle cells) [27], suggesting the identification of a multipotent precursor that is not restricted to the cardiac muscle lineage.

It has long been appreciated that hESCs differentiate into a heterogeneous population of atrial, ventricular and specialized conduction CMs in culture [6], [7], [8], [9], [10]. Whether this reflects a stochastic process *in vitro*, and is driven by a combination of genetic programming and the extracellular milieu *in vivo*, has not been established. Understanding the mechanisms that drive CM subtype specification, however, will be essential to both understanding cardiac development and developing cell-based reagents for myocardial therapy.

In summary, we have identified multipotent human myocardial precursors (hMPs) using an αMHC-GFP reporter hESC line. We have demonstrated that reporter activation is restricted to hESC-derived CMs differentiated *in vitro* and *in vivo*, and that the reporter does not interfere with hESC genomic stability. Importantly, we show that hMPs give rise to multiple CM subtypes and can be used to explore CM differentiation on the molecular level by expression profiling. These precursors will provide important insight into the pathways regulating human myocardial development, and provide a novel therapeutic approach to stem cell therapy for cardiac disease.

## Materials and Methods

### hESC culture and differentiation

All work with hESCs was done with the approval of the UCSF Stem Cell Research Oversight Committee. The parent H9 hESC line (WA09; WiCell) was maintained on irradiated mouse embryonic fibroblast (MEF) feeder cells (Millipore) in a medium comprised of Knockout DMEM (Invitrogen) supplemented with 20% Knockout Serum Replacement (Invitrogen), 2 mM glutamine, 0.1 mM nonessential amino acids, 0.1 mM β-mercaptoethanol and 15 ng/ml recombinant human FGF-basic (R&D Systems). Differentiation was initiated by human embryoid body (hEB) formation in suspension as previously described [28]. Briefly, colonies of hESCs were dissociated into small clusters by exposure to Collagenase IV (Sigma-Aldrich), then allowed to differentiate in a medium comprised of Knockout DMEM (Invitrogen) supplemented with 20% Defined Fetal Bovine Serum (Hyclone), 2 mM glutamine, 0.1 mM non-essential amino acids, and 0.1 mM β-mercaptoethanol. After 4–7 days in suspension, hEBs were attached to gelatin-coated 12-well culture plates and allowed to differentiate for an additional 14–21 days. For re-culture and expression profiling experiments, hEBs were dissociated with TrypLE Express (Invitrogen) to generate single cell suspensions, stained with propidium iodide to distinguish between live and dead cells, and sorted on the basis of GFP expression using a FACSAria (Becton Dickinson) with standard filter sets using previously described methods [29], [30].

### Plasmid and cell line construction

The 2K7_bsd_ lentivector (kindly provided by David Suter [14]) was used to assemble a lentiviral plasmid capable of driving the expression of enhanced green fluorescent protein (GFP) by a ubiquitin-C or α-myosin heavy chain (αMHC) promoter. The human ubiquitin-C promoter (position −1225 to −6 upstream from the translation start site; [31]), amplified by PCR, or a 1.7 kb *Eco*RI-*Sal*I fragment from the mouse αMHC promoter (generously provided by Jeffrey Robbins [13]) was inserted into the pENTR 5′-TOPO entry vector using the pENTR 5′-TOPO TA Cloning Kit (Invitrogen). A cDNA encoding GFP was inserted into the pENTR/D-TOPO entry vector using the pENTR/D-TOPO Cloning Kit (Invitrogen). The promoters and reporter gene were inserted into the double recombination site of the 2K7_bsd_ plasmid using LR Clonase Plus Enzyme Mix (Invitrogen) according to the manufacturer's instructions.

### Immunocytochemistry

The lineage fate of differentiated hEBs was determined by staining 14-day-old, adherent differentiating hEBs attached to cover slips in 12-well culture plates. Cover slips were fixed with 4% paraformaldehyde, then permeabilized with 50% methanol/50% PBS, then 100% methanol, then 50% methanol/50% PBS/0.1% Triton X-100, and finally PBS/0.1% Triton X-100. hEBs were incubated with blocking buffer (PBS/10% horse serum/1% BSA/0.1% Triton X-100), then with primary antibody (1–5 µg/ml) in blocking buffer. Primary antibodies used were mouse anti-human cardiac troponin T (LabVision/Neomarkers MS-295-P1; clone 13–11), mouse anti-human cardiac α-actinin (Sigma A7732; clone EA-53), mouse anti-human alpha-fetoprotein (Sigma A8452; clone C3), mouse anti-human nestin (R&D Systems MAB1259; clone 196908) or mouse anti-human smooth muscle actin (R&D Systems MAB1420; clone 1A4). Cover slips were washed with blocking buffer, incubated with 1∶500 dilution of goat anti-mouse Alexa-Fluor 594 (Invitrogen A20185), washed with PBS/1% Triton X-100, then mounted with PBS and DAPI and analyzed by confocal microscopy using a Zeiss LSM510 META system. Alternatively, 8-day-old differentiating hEBs were sorted for GFP expression and re-cultured in differentiation medium in the presence of 10 µM p160 Rho-associated coiled-coil kinase inhibitor (Calbiochem 688000; Y-27632). At 14 days, differentiating hEBs were stained *in situ* in culture plates as described above using the same antibodies, and analyzed by immunofluorescence microscopy using a Nikon Microphot-FXA fluorescence/phase microscope and QImaging Retiga 2000R digital camera (Diagnostic Instruments) with MetaMorph software (Molecular Devices).

### Teratomas

All experiments involving animals were done with the approval of the UCSF Institutional Animal Care and Use Committee. To form teratomas, 5×10^5^ hESCs were mixed with an equal volume of 1 mg/ml *Phaseolus vulgaris* lectin (PHA-P L1668; Sigma), pelleted, and incubated in growth medium overnight at 37°C, 5% CO_2_ in a 0.4 µm MILLICELL (Millipore). At least 2 cell pellets were grafted under each kidney capsule of 8-week-old female CB17 SCID-Beige mice (n = 7) using published techniques [32]. Transplanted cells formed teratomas in the recipients and were analyzed 10 weeks after grafting. Teratomas were fixed in 10% buffered formalin, embedded in paraffin, and 5 µm sections were stained with purified polyclonal rabbit anti-GFP (Molecular Probes A11120) at 1∶1500 and biotinylated goat anti-rabbit IgG (Vector BA-1000). Slides were developed using the VECTASTAIN Elite ABC kit (Vector) and counterstained with hematoxylin and eosin to identify tissue structures.

### Quantitative real-time PCR

For analysis of transcript expression, GFP^+^ hESCs were sorted by FACS at indicated time points, or beating areas from approximately 20 hEBs were visualized with a Leica MZ6 microscope and manually excised using an 18 g needle. The collected tissue samples were treated with 0.05% Trypsin-EDTA to generate a single cell suspension prior to RNA isolation. RNA was isolated and cDNA synthesized from ∼50,000 hEB-derived cells or proliferating hESCs using the Taqman Gene Expression Cells-to-CT kit (Ambion). cDNA was quantitated using a Nanodrop ND-1000 Spectrophotometer (Nanodrop Technologies, ND Software version 3.3.0). Linear pre-amplification of target sequences was accomplished using the Applied Biosystems PreAmp system. Relative expression was determined using the TaqMan Assay (Applied Biosystems) on an ABI 7300 Real-Time PCR system with the following primer pairs (ABI): GATA4 (Hs00171403_m1), NKX2-5 (Hs00231763_m1), Is~l1Hs 01099687_m1;Hs00158126_m1sarcolipin (Hs00161903_m1;Hs01888464_s1), HCN4 (Hs00975492_m1;Hs00175760_m1), MLC2a (Hs00221909_m1), MLC2v (Hs00166405_m1;Hs01125721_m1), αMHC (Hs00411908_m1), cTnT (Hs00165960_m1), smooth muscle actin (Hs00242273_m1), α-fetoprotein (Hs00173490_m1), nestin (Hs00707120_s1), α1 integrin (Hs00235030_m1), α2 integrin (Hs00158148_m1), α4 integrin (Hs00168433_m1), α5 integrin (Hs00233732_m1), α6 integrin (Hs01041011_m1), α7 integrin (Hs00174397_m1), αv integrin (Hs00233790_m1), β1 integrin (Hs00559595_m1), β5 integrin (Hs00609896_m1), and GAPDH (4326317E). Cycle times to detection were normalized against a reference gene, GAPDH, and relative changes were calculated using ABI Version 1.4 Sequence Detection Software.

### Microelectrode array analysis

Individual GFP-expressing and wild type hEBs demonstrating contractile activity were mechanically dissected and plated on fibronectin-coated microelectrode arrays (MEA; Multi Channel Systems, Reutlingen, Germany). Mouse atrial HL-1 cells (generously provided by William Claycomb, LSU Health Sciences Center) were cultured on MEAs as control. The MEA system consisted of a 50×50 mm glass substrate with an embedded 1.4×1.4 mm matrix of 60 titanium nitride-gold contact electrodes with interelectrode distance of 200 µm. This allowed for simultaneous recording of extracellular field potentials (FP) from all electrodes over extended periods of time. Spontaneous electrical activity was recorded at 10 kHz. The temperature was kept at 37°C. Analysis of recordings was done using MC_Rack (Multi-Channel Systems) and a customized toolbox for MATLAB. The following parameters were determined: size of the largest negative peak (FP_min_), last postive peak of the cycle (FP_max_), the time interval between FP_min_ and FP_max_ (FP_dur_), and decay of extracellular potential (time from the onset of the FP to FP_min_ = FP_rise_) [19], [20].

### Array comparative genomic hybridization

Genomic DNA (300–500 ng) from the αMHC-GFP reporter hESC line was isolated using the Qiaeasy DNA kit for tissue (Qiagen). DNA was labeled with Cy3 and Cy5 using the BioPrime DNA labeling system (Invitrogen), hybridized to HumArray 3.2 Human chromosome arrays, and analyzed for chromosomal composition. Differentially labeled human male reference genomic DNA was run as a control. The HumArray 3.2 array contains 2,464 bacterial artificial chromosome clones spotted in triplicate and distributed uniformly across the genome. Each clone contains at least one STS and is mapped to the human genome sequence. Clones containing unique sequences near the telomeres and genes known to be significant in cancer and medical genetics are included on these arrays.

### mRNA expression profiling

Sample preparation, labeling, and array hybridizations were performed according to standard protocols from the UCSF Shared Microarray Core Facilities and Agilent Technologies (http://www.arrays.ucsf.edu and http://www.agilent.com). Total RNA quality was assessed using a Pico Chip on an Agilent 2100 Bioanalyzer (Agilent Technologies). RNA was amplified using the Sigma whole transcriptome amplification kit following the manufacturer's protocol (Sigma-Aldrich), and subsequent Cy3-CTP labeling was performed using the NimbleGen one-color labeling kit (Roche-NimbleGen). The size distribution and quantity of the amplified product was assessed using an Agilent 2100 Bioanalyzer and a Nanodrop ND-8000 (Nanodrop Technologies); the labeled DNA was assessed using the Nandrop 8000, and equal amounts of Cy3 labeled target were hybridized to Agilent human whole genome 4×44 K Ink-jet arrays. Hybridizations were performed for 14 hrs, according to the manufacturers protocol. Arrays were scanned using an Agilent microarray scanner and raw signal intensities were extracted with Feature Extraction v.10.1 software (Agilent Technologies). Data were further analyzed using Ingenuity Pathways Analysis (Ingenuity® Systems) to identify biological pathways involved in CM differentiation. The false discovery rate (FDR) from the data set was used for canonical pathways analysis.

### Statistics

Student's t-test was used to compare the means of the frequency count distributions of FP_dur_ and FP_rise_. A value of p<0.05 was considered significant. These analyses were performed using SPSS v.16 (SPSS, Inc.) for Macintosh.

The mRNA expression array dataset was normalized using the quantile normalization method [33]. No background subtraction was performed, and the median feature pixel intensity was used as the raw signal before normalization. A one-way ANOVA linear model was fit to the comparison to estimate the mean M values and calculated FDR for each gene for the comparison of interest. All procedures were carried out using functions in the R package *limma* in Bioconductor [34], [35].

## Supporting Information

Movie S1 **Sorted αMHC-GFP^+^ hESCs form beating CMs** ***in vitro*** **.** αMHC-GFP hESCs were cultured under differentiation conditions for 8 days, suspended as single cells and sorted for GFP expression. GFP^+^ cells were re-cultured under differentiation conditions for an additional 6 days, then analyzed *in situ* by phase contrast video microscopy. A typical culture demonstrating multiple foci of spontaneous contractile activity is shown. Magnification, 10X.
(MOV)

Movie S2 **Sorted αMHC-GFP^+^ hESCs form beating CMs** ***in vitro*** **.** Conditions as described for Movie S1. A typical culture demonstrating multiple foci of spontaneous contractile activity is shown at lower magnification. Magnification, 4X.
(MOV)
